# Features of the performance exposure in girls involved in cyclic and acyclic sports

**DOI:** 10.25122/jml-2020-0054

**Published:** 2021

**Authors:** Timur Cherkesov, Cherim Ingushev, Anna Konopleva, Radmir Cherkessov, Magomed Gairbekov, Azamat Zhukov

**Affiliations:** 1. Department of Theory and Technology of Physical Culture and Sports, Institute of Pedagogy, Psychology and Physical Education and Sports Education, Federal State Budgetary Educational Institution of Higher Education “Kabardino-Balkarian State University named after H.M. Berbekov”, Kabardino-Balkaria Republic, Russian Federation; 2. Department of Emergency Situations, Institute of Pedagogy, Psychology and Physical Education and Sports Education, Federal State Budgetary Educational Institution of Higher Education “Kabardino-Balkarian State University named after H.M. Berbekov”, Kabardino-Balkaria Republic, Russian Federation; 3. North-Caucasian Institute for Advanced Studies, Krasnodar University of the Ministry of Internal Affairs of Russia, Kabardino-Balkaria Republic, Russian Federation; 4. Department of Physical Education, Federal State Budgetary Educational Institution of Higher Education “Dagestan State University”, Republic of Dagestan, Russian Federation

**Keywords:** physical performance, cyclic and acyclic sports, heart rate monitoring, blood pressure, cardiovascular system, ANS – autonomic nervous system, BP – blood pressure, CNS – central nervous system, CVS – cardiovascular system, HR – heart rate, MOC – maximum oxygen consumption, PR – pulse rate

## Abstract

According to the definition adopted by the international biological program, physical performance is characterized by maximum oxygen consumption (MOC). Profession, lifestyle, and sport affect the value of the MOC. For anatomy and physiology, oxygen delivery to muscle tissue depends on the state of the respiratory and cardiovascular system, the amount and composition of blood. In this case, the leading role belongs to the cardiac activity, namely to the magnitude of the shock and minute volumes of blood in working conditions. High values of MOC and, consequently, a large work capacity are characteristic of athletes specializing in cyclic sports with moderate and high power. The purpose of the research was to evaluate the adaptive response of the cardiovascular system of girls involved in cyclic and acyclic sports as well as those not involved in sports. The study was conducted in 2018. During the research, we used pulse oximetry and determination of blood pressure according to N.S. Korotkov, as well as an assessment of the adaptation of the cardiovascular system according to the Ruffier Index. Studies have shown differences in the level of performance of girls involved in cyclic sports (athletics) and acyclic sports (karate and taekwondo), as well as non-sports. During the experiments, heart rate and blood pressure indicators were recorded at rest and after exercise, and the Ruffier Index, which reflects the level of performance of the participants, was calculated.

## Introduction

The pattern of cyclic movements is the following: all phases of movements that exist in one cycle are present in other cycles in the same sequence. The cycles are inseparable, and the end of the exercise is the beginning of the next movement.

The role of the physiological basis of cyclic movements is performed by the rhythmic motor chain reflex, which has an unconditionally reflective origin and is maintained automatically. A significant part of cyclic movements are natural locomotions (movements) or are based on them. The main variables in cyclic movements are the power and duration of the exercise performed. Power is determined by the frequency of motion cycles, amplitude, and strength of movements.

Dependence of the maximum duration of exercise on its power or speed of movement is common to all cyclic movements.

Currently, in the context of the increased interest of millions of people of various ages in physical exercises and sports, the urgency of the problem of adapting the cardiovascular system (CVS) to physical activity concerns specialists working in the field of sports physiology and clinicians. The beneficial effects of rational physical training on the body and its CVS are well known.

The circulatory system is involved in all manifestations of the body’s vital activity, providing adequate delivery of oxygen and nutrients, as well as the timely removal of metabolic products. Moreover, with its multi-level regulation, CVS is a functional system, which ensures a given level of the whole functioning organism. Other things being equal, we can assume that the given level of functioning of the whole organism corresponds to the equivalent level of functioning of the circulatory apparatus. Moreover, the regulation of hemodynamics is closely related to all levels of control of the physiological functions of the body, including the central nervous system (CNS), the autonomic nervous system (ANS), and the hypothalamus-pituitary-adrenal axis. In this regard, by studying the management processes of CVS, one can judge the state of the systems. Therefore, the adaptive changes that occur in the cardiovascular system during physical training should be under constant medical supervision, and deviations in the state of health and violation of the cardiovascular reactions, which is manifested in all people differently, should become the object of close attention of specialists for the development of methods for early diagnosis, the correct clinical assessment, the selection of rational and effective preventive and treatment measures. Numerous studies carried out in this regard do not fully reveal the mechanisms and ambiguously explain the specifics of cardiohemodynamic response to physical activity. A significant role in inhibiting or enhancing reactions in response to various stressful effects, including physical exertion, is played by “determining” factors – gender, age, genetic predisposition, typological features, and others [4].

The question about the possibility of using the concept of circulatory types to assess the functional state of CVS and the characteristics of its response under the influence of physical activity remains insufficiently explored.

It is known that when some actions are performed, after some time, a person starts to feel that it is becoming challenging to carry them out. Externally, this can be determined by several visible signs, such as the tension of the facial muscles and the appearance of perspiration.

At the same time, more significant physiological changes occur in the body. However, despite this, due to volitional efforts, a person can maintain the intensity of work for some time. This condition is called the compensated fatigue phase.

However, if continuing to perform the given exercise, a decrease in its intensity appears, despite the manifested volitional efforts – the decompensated fatigue phase sets in. Fatigue is usually called a temporary decrease in performance caused by work. If we offer the same exercise to several people, it can be noted that their fatigue does not occur simultaneously. The reason for this is a different degree of development of working performance.

Depending on the specifics of activity types, mental, emotional, and physical fatigue are distinguished. Although all of the above types of fatigue are represented in all activities, to a greater or lesser extent, the sphere of physical education, physical fatigue caused by muscular activity, and the working performance developing in the process of overcoming it are of importance [5–8].

According to the definition adopted by the international biological program [9], physical performance is characterized by maximum oxygen consumption (MOC).

Profession, lifestyle, sport affect the MOC value. For anatomy and physiology, oxygen delivery to muscle tissue depends on the state of the respiratory and cardiovascular system, the amount and composition of blood. The leading role, in this case, belongs to the cardiac activity, namely the magnitude of the shock and minute volumes of blood in working conditions. High MOC values and, consequently, significant working performance is characteristic of athletes specializing in cyclic sports with moderate and high power [9–13].

It is indisputable that aerobic exercises for the development of working performance are necessary for all athletes and are extremely necessary for representatives of those professions which involve the factor of physical inactivity.

In this regard, knowledge of the physiological foundations of working performance and the means and methods contributing to its increase in each particular case acquire special significance.

Based on the above mentioned, it can be confirmed that the topic of this work, devoted to the study of the development level of the working performance of 17–18-year-old girls engaged in cyclic and acyclic sports and those not involved in sports, is relevant.

The object of research is the performance as the basis for the development of physical qualities.

The research subject is the peculiarities of the development of athletes’ performance in cyclic and acyclic sports. The purpose of the research was to evaluate the adaptive response of the cardiovascular system of girls involved in cyclic, acyclic sports and those not involved in sports.

The scientific novelty of this scientific research lies in the implementation of an integrated approach to study the characteristics of the manifestation of working performance in girls involved in cyclic and acyclic sports using a pulsometer and determining blood pressure according to N.S. Korotkov, as well as assessing the adaptation of the cardiovascular system according to the Rufier Index [14–19].

The comprehensive behavior of this comparative analysis made it possible to obtain the most reliable and scientifically based data on determining the characteristics of the manifestation of working performance in girls involved in cyclic and acyclic sports [20–24].

It was necessary to solve the following problem: to conduct a comparative analysis of the working performance of girls who:

1. Do athletics (types of running exercises);
2. Do martial arts;
3. Do not play any sports.

The hypothesis of the research was the assumption that cyclic sports are more effective for increasing the overall performance of the body compared to acyclic sports and physical education according to the program of higher educational institutions.

## Material and Methods

Thirty athletes of the Athletics Department of the Specialized Youth Sports School of the Olympic Reserve (Nalchik), 30 athletes specializing in karate and taekwondo at the State Department of the Youth Sports Center of the Educational and Science Ministry of Kabardino-Balkaria Republic and 30 second-year students of the Law Faculty of Kabardino-Balkarian State University named after H.M. Berbekov» (Football Club) took part in our study.

Qualifications of athletics:

• Master of Sports in the Russian Federation– 3 people;
• Candidates for Master of Sports in the Russian Federation – 3 people;
• 1^st^ category – 5 people;
• 2^nd^ category – 7 people.

Those involved in karate and taekwondo:

• Master of Sports in the Russian Federation– 2 people;
• Candidates for Master of Sports in the Russian Federation – 4 people;
• 1^st^ category – 5 people;
• 2^nd^ category – 6 people.

A group of girls engaged in physical education only in academic studies did not have a sports category.

The investigation was conducted at the educational and research laboratory “Biotechnology” of the Faculty of Physical Culture and Sports of KBSU. Testing was carried out according to the pulse rate (PR) per minute: at rest, after physical activity – 30 deep squats for 30 s, then 1 minute after the exercise. We used the Polar FT4M cardio monitor to estimate the heart rate [25–27]. We used the data obtained to evaluate the adaptation of the cardiovascular system according to the Ruthier Index. Also, at rest and after exercise, blood pressure (BP) was measured according to N.S. Korotkov: systolic (BPs), diastolic (BPd), calculated pulse pressure (BPp), and Robinson index.

## Results

**Figure 1.**
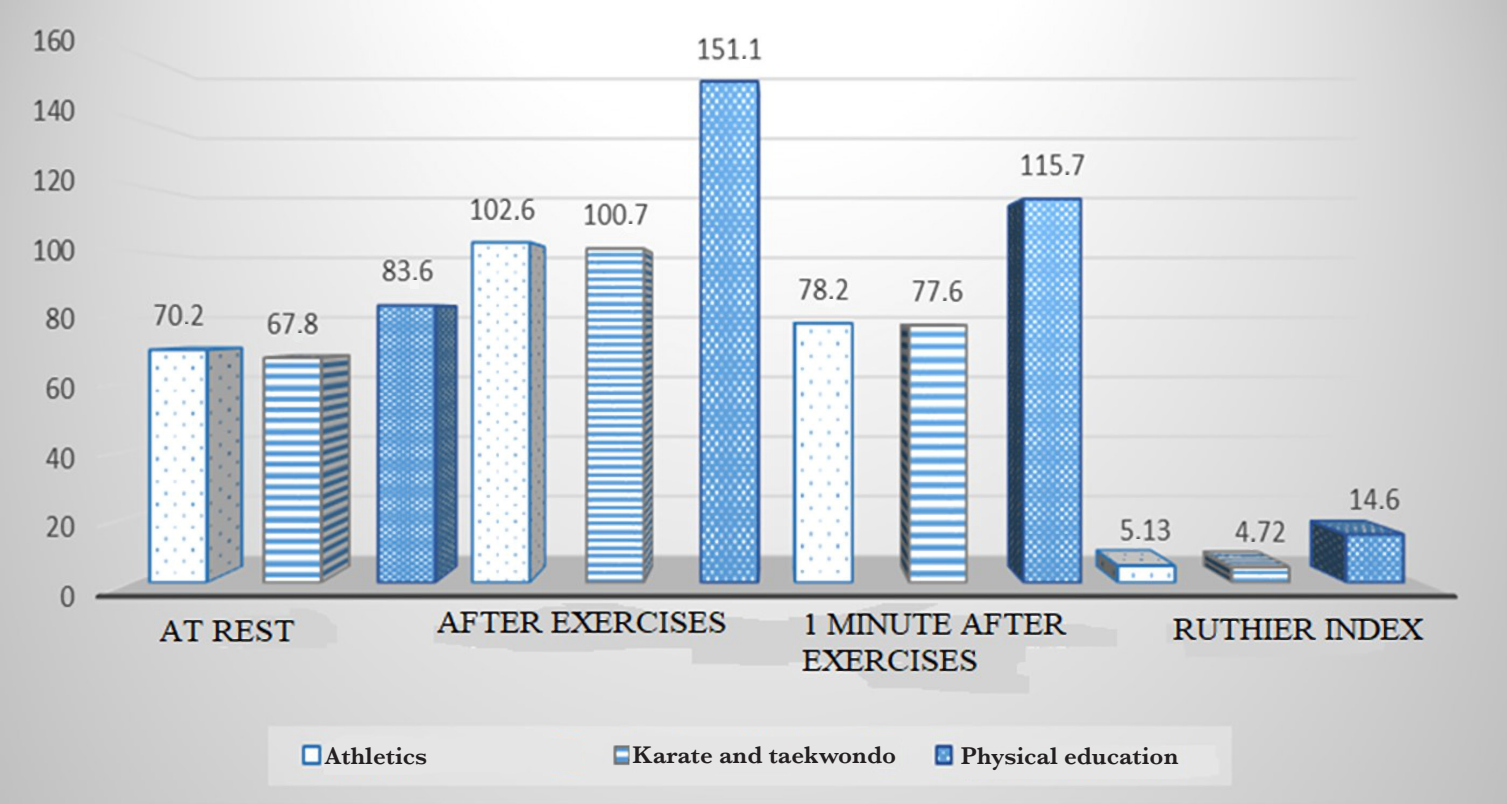
Heart rate and Ruffier Index.

As a result of the research, the following graph was revealed (Figure 1). At rest, the pulse rate of athletes was 70.2±2.02 beats/min; one hour after loading – 102.6±2.36 bpm, 1 minute after loading – 78.2±2.69 bpm. For those practicing karate and taekwondo – 67.8±2.69 beats/min., 100.7±3.37 beats/min, and 77.6±2.36 bpm, respectively. In girls engaged in physical exercises only during academic classes in physical education, these indicators were 83.6±2.69 beats/min., 151.1±4.38 beats/min, and 115.7±2.02 bpm, respectively.

Thus, the pulse rate in response to physical activity increased by 46% in athletes, by 48.5% in those practicing martial arts, and by 80.7% in non-athletes. An increase in heart rate by 25–50% indicates a satisfactory condition of the cardiovascular system.

In the recovery period, 1 minute after the exercises, athletes had a heart rate of 11.4% higher than usual at rest, 14.5% in martial artists, and 38.4% in non-athletes. According to the heart rate at rest and after exercise, the differences between the groups of athletes and karate and taekwondo women are unreliable (P>0.05). Girls who are not involved in sports were significantly inferior to athletes regarding this indicator (P<0.05).

**Figure 2.**
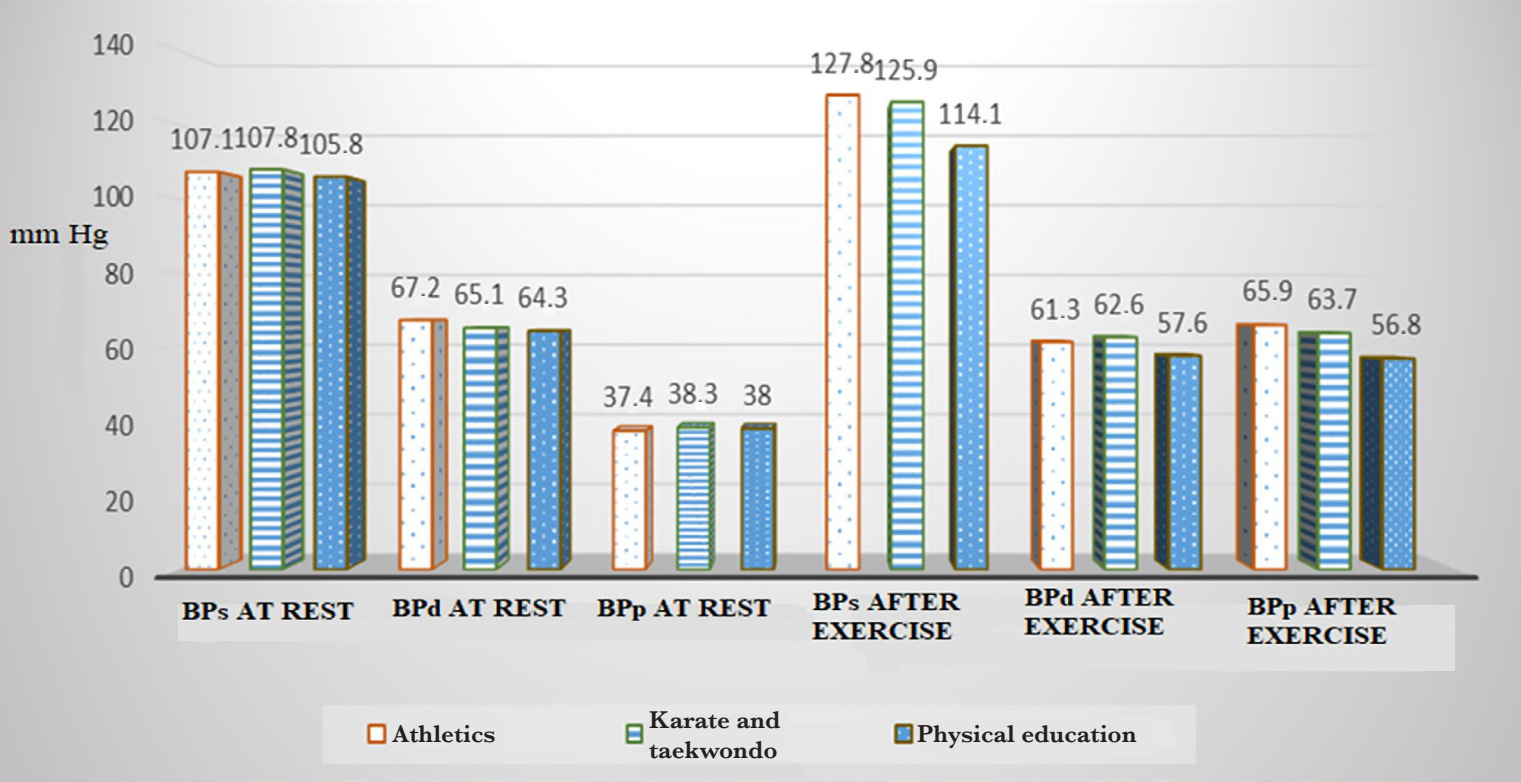
Dynamics of blood pressure. BPs – systolic blood pressure; BPd – diastolic blood pressure; BPp – pulse blood pressure.

In athletes, systolic blood pressure was 107.1±1.68 mmHg, diastolic blood pressure was 67.2±3.37 mmHg, and pulse pressure was 37.4±2.69 mmHg. During the exercises, systolic blood pressure increased by 20.7 mmHg, diastolic blood pressure decreased by 5.9 mmHg, and blood pressure increased by 28.5 mmHg (Figure 2).

For girls involved in karate and taekwondo, these indices at rest are respectively equal: 107.8±2.36 mmHg, 65.1±4.04 mmHg, and 38.3±2.02 mmHg. During the exercises, systolic blood pressure increased by 18.1 mmHg, diastolic blood pressure decreased by 2.5 mmHg, and pulse pressure increased by 25.4 mmHg.

The response to the exercises in these cases of comparison is eutonic, in contrast to the group of subjects not involved in sports, where the systolic blood pressure was 105.8±4.04 mmHg, the diastolic blood pressure was 64.3±3.36 mmHg and pulse pressure was 38.0±2.02 mmHg. During the exercises, systolic blood pressure increased by 8.3 mmHg, diastolic blood pressure decreased by 6.7 mmHg, and pulse pressure increased by 18.8 mmHg. It is an inadequate response to the exercises.

Considering that pulse pressure is an indirect indicator of systolic blood volume, it can be argued that athletes’ heart at rest works more economically, the adaptation exercise is higher for a test than martial artists (in a group of athletes at rest, the pulse pressure is lower, and higher than the group engaged in karate and taekwondo after exercise).

In the third group of subjects, the efficiency of heart work at rest practically does not differ from the group of karate and taekwondo athlete-girls, and the load on adaptation is significantly lower than for martial arts and athletes. The difference between athletes is much higher.

The Ruthier index was 5.13±0.44 for athletes, 4.72±0.37 for martial artists, and 14.6±1.65 for girls who are not involved in sports. It can be argued that both athletes and karate and taekwondo women have a normal functional state of CVS, while non-athletes have an unsatisfactory functional state of CVS.

## Discussion

As a result of research to assess the adaptive and functional capabilities of the cardiovascular system in girls specializing in athletics, martial arts, it was found that the level of blood pressure, heart rate (HR), and the Ruthier index are almost identical.

A higher (although not significantly different) heart rate in response to the exercise in the group of athletes may be a sign of higher aerobic endurance, which is typical for those involved in cyclic sports.

On the other hand, such an insignificant advantage of athletes over martial arts, in our opinion, is due to two reasons:

• Both groups include highly qualified athletes;
• Athletes (according to the trainer) are less active in enhancing sportsmanship.

Greater profitability of the heart work in athletes compared with those not involved in sports is observed not only when performing standard work (as recorded in our studies) but also in cases where the exercise increases significantly and is expressed as a percentage of the maximum individual oxygen consumption, as stated by Platonov [5]. It indicates not only increased oxygen delivery to the muscles but also its more efficient utilization in the muscles.

The ability to perform work with a high percentage of oxygen consumption of the maximum volume level (max VO2) without significant accumulation of lactic acid in the blood is an essential factor determining the level of development of endurance.

## Conclusion

In the process of the research, we revealed that:

• The reaction to the exercises in the groups of girls-athletes is eutonic, in contrast to the group of subjects not involved in sports: the systolic pressure indicators are respectively equal: 127.8±2.69, 125.9±2.37 and 114.1±4.04 mmHg;
• The heart of athletes works most efficiently – the pulse pressure indicators (although unreliable, P> 0.05) differ from the pulse pressure indicators of martial artists – lower at rest, and higher after the exercise compared to girls not involved in sports; the difference in these indicators was very significant (P<0.05);
• The functional state of the cardiovascular system in athletes and karate and taekwondo athletes is normal; in non-athletes, it was unsatisfactory: the Ruffier index was 5.13±0.44, 4.72±0.37 and 14.6±1.65, respectively. The indicator of the third group of subjects (P<0.05) had a significant difference;
• The low-performance indicators of girls engaged only in physical education classes indicate a low efficiency of the classes in terms of weekly workload and methods of conducting a particular lesson.

The increase in motion activity has a positive effect on the performance of athletes, increases the functionality of the cardiovascular system, and, accordingly, the whole organism. Minor changes in indicators such as heart rate and cardiac contractility indicate higher functional capabilities of the athletes’ cardiovascular system during long exercises.

## Acknowledgments

### Consent to participate

Informed written consent was collected from the participants after the study objectives were explained.

### Conflict of interest

The authors declare that there is no conflict of interest.
